# ClermonTyping: an easy-to-use and accurate *in silico* method for *Escherichia* genus strain phylotyping

**DOI:** 10.1099/mgen.0.000192

**Published:** 2018-06-19

**Authors:** Johann Beghain, Antoine Bridier-Nahmias, Hervé Le Nagard, Erick Denamur, Olivier Clermont

**Affiliations:** ^1^​ IAME, UMR 1137, INSERM, Université Paris Diderot, Sorbonne Paris Cité, F-75018 Paris, France; ^2^​ Assistance Publique-Hôpitaux de Paris, Hôpital Bichat, Laboratoire de Génétique Moléculaire, F-75018 Paris, France

**Keywords:** epidemiology, phylogeny, *E. coli*, *Escherichia*, typing, tool

## Abstract

The genus *Escherichia* is composed of *Escherichia albertii*, *E. fergusonii*, five cryptic *Escherichia* clades and *E. coli sensu stricto.* Furthermore, the *E. coli* species can be divided into seven main phylogroups termed A, B1, B2, C, D, E and F. As specific lifestyles and/or hosts can be attributed to these species/phylogroups, their identification is meaningful for epidemiological studies. Classical phenotypic tests fail to identify non-*sensu stricto E. coli* as well as phylogroups. Clermont and colleagues have developed PCR assays that allow the identification of most of these species/phylogroups, the triplex/quadruplex PCR for *E. coli* phylogroup determination being the most popular. With the growing availability of whole genome sequences, we have developed the ClermonTyping method and its associated web-interface, the ClermonTyper, that allows a given strain sequence to be assigned to *E. albertii*, *E. fergusonii*, *Escherichia* clades I–V, *E. coli sensu stricto* as well as to the seven main *E. coli* phylogroups. The ClermonTyping is based on the concept of *in vitro* PCR assays and maintains the principles of ease of use and speed that prevailed during the development of the *in vitro* assays. This *in silico* approach shows 99.4 % concordance with the *in vitro* PCR assays and 98.8 % with the Mash genome-clustering tool. The very few discrepancies result from various errors occurring mainly from horizontal gene transfers or SNPs in the primers. We propose the ClermonTyper as a freely available resource to the scientific community at: http://clermontyping.iame-research.center/.

## Data Summary

The code used in the methods described here is deposited in a github repository at the following address: https://github.com/A-BN/ClermonTyping

We confirm all supporting data, code and protocols have been provided within the article or through supplementary data files. We provide three supplementary tables. This material and corresponding links to the files are available for download in the online version of this article (Supplementary Material).

**Impact Statement**

The identification of *Escherichia* species members, except *Escherichia coli sensu stricto*, as well as of the *E. coli* phylogroups is difficult using classical phenotypic data. Such species/phylogroup identification is nevertheless highly meaningful in epidemiological studies, as an association has been observed between the species/phylogroup and lifestyle. The scientific community largely uses PCR assays developed by Clermont and colleagues that allow rapid and easy species/phylogroup identification. With the development of high-throughput sequencing technologies that has as a corollary an increasing number of whole genomes sequences, we have developed and validated the ClermonTyping method and its associated web-interface, the ClermonTyper, which is based on the concept of *in vitro* PCR assays and maintains the principles of ease of use and speed. This *in silico* method allows a given strain sequence to be assigned to *E. albertii, E. fergusonii, Escherichia* clades I–V, *E. coli sensu stricto* as well as to the seven main *E. coli* phylogroups. We propose the ClermonTyper as a user-friendly and very efficient tool for *Escherichia* species/phylogroup identification and provide it freely available to the scientific community.

## Introduction

The genus *Escherichia* is composed of *Escherichia albertii*, *E. fergusonii*, five cryptic *Escherichia* clades (I–V) and *E. coli* [1]. Based on average nucleotide identity of 95 % to define a species, *E.* clade I should be considered as a subspecies of *E. coli*; *E.* clades III and IV as subspecies of a novel species and *E.* clades II and V as two novel species [2–4]. However, we here use the nomenclature of *E.* clades I–V and refer to the classical *E. coli* as *E. coli sensu stricto. E. albertii* is the most divergent species of the genus whereas *E. fergusonii* is closely related to *E. coli sensu stricto* [1]. Classical phenotypic tests such as API 20 *Enterobacteriaceae* (bioMérieux) or MALDI-TOF mass spectrometry fail to accurately identify the non-*E. coli sensu stricto* species. The cryptic clades are phenotypically indistinguishable from classical *E. coli* [3]. Furthermore, the species *E. coli* can be divided into seven main phylogroups termed A, B1, B2, C, D, E and F [5]. Interestingly, specific lifestyles and/or hosts can be attributed to these species/phylogroups [6–8] and the assignment of a given strain to such species/phylogroups is meaningful and classically performed in epidemiological studies. Multilocus sequence typing (MLST) using either the Warwick [9] or the Pasteur Institute [10] scheme provides complementary and useful information as it allows us to characterize strains further into clonal complexes and sequence types.

Clermont and colleagues have since 2000 provided several PCR assays allowing the easy and rapid assignment of the strains in these species/phylogroups. One of the most popular is the triplex [11], becoming quadruplex in 2013 [5], Clermont PCR, which assigns *E. coli sensu stricto* strains to four and seven phylogroups, respectively. PCR assays for *Escherichia* clade assignment [1] and more recently for *E. albertii* [12, 13] and *E. fergusonii* [13] assignment have also been reported.

With the growing number of available complete genomes (more than 10 000 *E. coli* genomes are available at the NCBI RefSeq database to date), it would be useful to have ‘*in silico* PCR assays’ that would allow us to assign strains to a specific species/phylogroup directly from the strain's complete sequence. Several groups have now reported such *in silico* Clermont phylo-typing but none of them provides precise methodology or a validation step [14–16]. In addition, one group has reported discrepant results between the core genome phylogenetic tree and the *in silico* Clermont typing for *E. coli* F phylogroup strains [16].

In this context, we have developed and validated the ClermonTyping method and its associated web-interface, the ClermonTyper, that allows us to assign a given strain sequence to *E. albertii*, *E. fergusonii*, *Escherichia* clades I–V, *E. coli sensu stricto* as well as to the seven main *E. coli* phylogroups. The ClermonTyping method is based on the concept of *in vitro* PCR assays and maintains the principles of ease of use and speed that prevailed during the development of the *in vitro* assays. We propose this tool as freely available to the scientific community.

## Theory and implementation

### General intention

The ClermonTyping is an *in silico* method that aims to reproduce the results of the *E. coli* phylo-typing by PCR proposed by Clermont *et al.* in 2000 [11] and updated in 2013 [5] as well as the various PCR assays developed to identify the *Escherichia* clades [1] and *E. albertii* [12]. In addition, we have developed in the present study an *E. fergusonii-*specific PCR. We designed the ClermonTyping with two main goals in mind: (i) it had to be fast and easy to use for all members of the scientific community anywhere and anytime, and (ii) the results had to be as concordant as possible with the *in vitro* method.

ClermonTyping comes in two flavours: a command line set of scripts and executables and a website, the ClermonTyper, with a very user-friendly interface that will allow anyone to assign/phylo-type *Escherichia* sp. strains with only a few clicks in a web browser.

### Outline of the method

The method takes a DNA FASTA/multi-FASTA formatted *Escherichia* sp. genome. A blast database is then created using this query genome and the BLASTn algorithm is called with specific parameters to find matches for a set of primers described in Table S1 (available in the online version of this article) [17]. The blast result is then interpreted as the PCR would be in terms of presence or absence of amplification for each pair of primers.

**Fig. 1.**
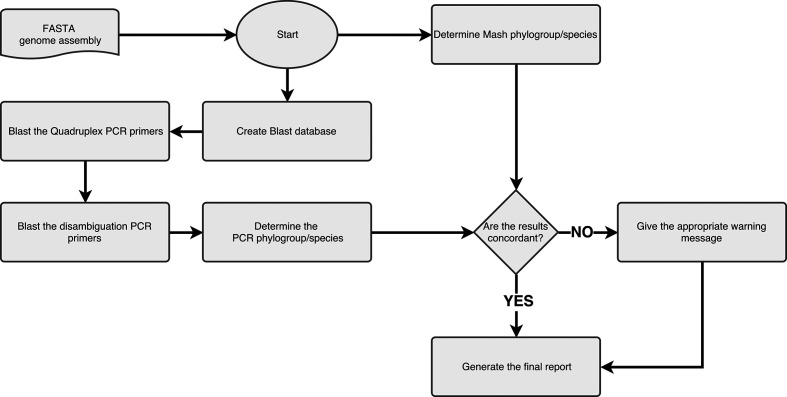
ClermonTyping method flowchart. The algorithm takes a FASTA file as input and determines the species/plylogroup in two distinct ways: by Mash and *in silico* PCR. A warning is given when the two methods do not reach a consensus.

The design of this PCR method can in some particular cases lead to misleading results (*in vitro* as well as *in silico*), for example when a SNP at a particular position prevents primer annealing or in cases of recombination or horizontal transfers, including insertion sequences (IS), between genomes [18]. To address these cases, we added a phylogroup determination step based on a genome-clustering tool called Mash [19]. Mash allows us to approximate a pairwise mutation distance between the query genome and a manually curated database representing as much as possible the *Escherichia* sp./phylogroup genomic diversity. We use a Mash2.0 screen command to estimate the nearest genome in an *Escherichia* sp. manually curated genomic database. What we term the ‘Mash group’ is the species/phylogroup of the nearest genome in the database (Fig. 1).

This information is then aggregated in the form of an HTML report easily interpretable for the user.

### Inputs and ouputs

#### Inputs

To run an analysis, the user will call the bash script clermonTyping.sh that accepts three arguments as described below.

The only mandatory input for ClermonTyping is a FASTA/multi-FASTA file containing assembled genomic DNA sequence(s) from *Escherichia* sp. The genome can be provided as a full chromosome (one contig) or as a set of multiple contigs. The sequence must be one of a single clone produced through any kind of sequencing method. The general quality of the sequence and its assembly is crucial for accurate results. This file path is passed to the script through the −−fasta argument.

The user can optionally input a name for the analysis using the −−name argument. The results are stored in a directory named after the −−name argument value (created inside the working directory). In the absence of a value, the results can be found in a directory named ‘analysis' followed by the current date as year, month, day and time: analysis_YYYY-MM-DD_hhmmss, e.g. analysis_2017_12_25_ 134557.

The third and last argument is also optional and, given through −−threshold, is an integer and sets the minimal size in nucleotides for a contig to be included in the analysis. This filter can be used to eliminate errors due to the presence of small contigs of poor quality in the assembly. The ideal threshold value will depend on each genome assembly but 2000, 1000 and 500 would be the most common figures to use. The default value is set to 0.

#### Outputs

ClermonTyping outputs multiple files, and we chose not to delete any intermediates that could be of interest for some users. The output directory will contain the following (in the case of no −−name given):

– db/; a directory containing the blast database

– analysis_YYYY-MM-DD_hhmmss.html; this file is the main report and contains most of the information pertaining to the analysis; it is also the form in which the online ClermonTyping will return its result, directly in the user's web browser

– analysis_YYYY-MM-DD_hhmmss.R; the R script that generated the html report

– analysis_YYYY-MM-DD_hhmmss.phylogroups.txt; a tab-separated value file written by clermont.py with the following fields: ‘fasta file name’, ‘obtained amplicons’, ‘quadruplex PCR results’, ‘supplementary PCR results’, ‘phylogroup’, ‘mash results filename’

– fasta_file_name.xml; an xml file written by blast n (one for each FASTA in the input query)

– fasta_file_name_mash_screen.tab; a tsv table written by Mash and described here [20] (one for each FASTA in the input query)

ClermonTyping also copies the query FASTA file into the output directory.

### *In silico* PCR assays

In order to mimic the behaviour of the *in vitro* PCR methods, the genomic FASTA file is first converted to a blast formatted database using the makeblastdb tool.

A set of 30 primers (15 primer pairs described in Table S1) is then fed to the blast n algorithm using the 90 % identity threshold and a word size of six [17]. The xml formatted blast report obtained is processed by a python script that translates it into a PCR result. Three conditions are required for a target to be considered a valid amplicon:

- forward and reverse primers must match on the same contig
- the amplicon size must not differ from its expected size by more than 20 % (allowing for small indels in the target)
- the three nucleotides located at the 3′ end of each primer must exhibit perfect homology with the matrix and there must not be more than six mismatches in the remainder of the primer annealing.

**Fig. 2.**
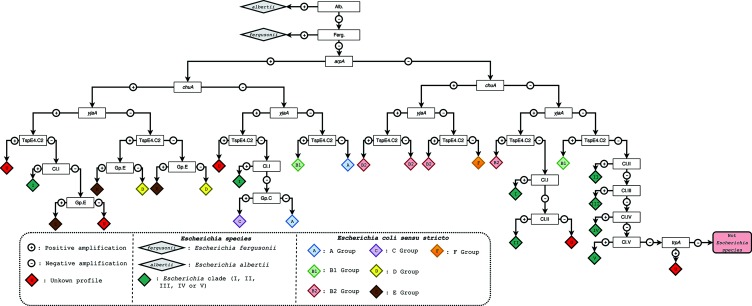
Complete flow scheme allowing species (*Escherichia albertii*, *E. fergusonii*, *E.* clades I–V and *E. coli*) and further *E. coli* phylogroup assignment used in the ClermonTyping method. The ClermonTyping method is based on the results of at least six PCR amplifications: an allele-specific amplification of *chuA* for *E. albertii* (Alb. at the top of the tree) and a specific amplification of *citP* for *E. fergusonii* (Ferg.) followed by *arpA*, *chuA* and *yjaA* and DNA fragment TSPE4.C2 for *E. coli sensu stricto* phylogroup assignment. Several additional amplifications may then be needed for a complete determination: allele-specific primers for phylogroups E and C (Gp.E and Gp.C, respectively) and allele-specific primers for *Escherichia* clades (Cl.I, Cl.II, Cl.III, Cl.IV and Cl.V). The amplification of *trpA* is used as a control for *Escherichia* species. The ‘Unknown’ profile will alert the user who will have to check the Mash assignation. A complete list of primers and targets is provided in Table S1.

The presence or absence of the different amplicons constitutes a profile that allows for species/phylogroup assignment (Fig. 2). The flow scheme starts with the *E. albertii* specific primers followed by the *E. fergusonii* primers developed in the present study. The different profiles for *E. coli sensu stricto* and *Escherichia* clades are respectively described by Clermont and colleagues in 2013 [5] and 2011 [1]. The possible returned profiles are: A, B1, B2, C, D, E, F, *E.* clade I, *E.* clade II, *E.* clade III, *E.* clade IV, *E.* clade V, *E. albertii* and *E. fergusonii*. Other profiles will simply be flagged as ‘Unknown’. They correspond to profiles never encountered *in vitro*. They can be due to various problems occurring during the sequencing process (strain contamination, poor quality sequence) or to horizontal gene transfers or SNPs in the primers. These profiles will alert the user who will have to check the Mash assignation.

### Mash species/phylogroup assignment

Although the quadruplex PCR method and associated PCR assays are very efficient and quickly and easily classify strains into *Escherichia* species/*E. coli* phylogroups, they can give erroneous results under some conditions. Indeed, by design the method only takes into account a very small proportion of the genome. A simple nucleotide variation, SNP or indel can completely prevent primer annealing and dramatically change the result given by the method. For example, a mutation in the most 3′ part of the region targeted by the primer TspE4.C2_F in a B1 strain would transform the result of the quadruplex PCR from phylogroup B1 (+ − − +) to phylogroup A (+ − − −). In the same way, any horizontal transfer encompassing one of the target DNA fragments would alter the result. There are many such possible cases.

In order to detect these particular cases, we take advantage of the efficiency and speed of the Mash genome distance estimation method [19]. Using this tool, we are able to determine the genomic relatedness of the query with all the strains present in the database and hence the most likely species/phylogroup to which it belongs.

### The Mash database

**Fig. 3.**
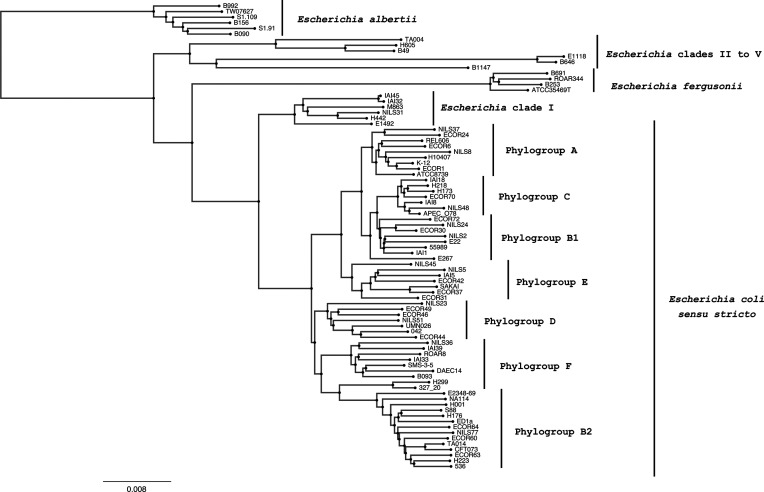
Neighbour-joining tree depicting the phylogenetic structure of the genus *Escherichia*. The distances are computed by Mash on the manually curated database containing 83 strains representing the *Escherichia* sp./phylogroup diversity. The tree is rooted on *E. albertii* strains, as they are the most divergent within the genus *Escherichia* [1]. Bar, 0.008 Mash distance unit.

The Mash database was created using the mash -sketch option and comprises 83 strains manually curated to represent much of the diversity of *E.* clades, *E. fergusonii* and *E. albertii* as well as representatives of *E. coli* phylogroups (Fig. 3). We chose not to include an extensive number of strains to keep it lightweight and very fast to interrogate.

These strains were then included as part of our testing dataset and will be discussed further below.

### Final report

The generation of the final report comprises a few validation steps that will allow printing of warning messages that are easily interpretable for the user. First, for each FASTA in the query, we analyse the Mash output and check that only one species/phylogroup stands out. Otherwise an output warning message is created indicating that the input FASTA file might contain multiple genomes. If Mash simply fails to find a close match in its database the most probable hypothesis is that the query FASTA is only a partial genome. As stated earlier, a mutation affecting the binding of one of the primers would lead to an incorrect species/phylogroup assignment. Mash is insensitive to these mutations and in the case of a discrepancy between the *in silico* PCR assay result and the Mash phylogroup/species the user will be warned and should be able to make a decision regarding the data.

### Implementation

The ClermonTyper web interface is hosted by CATIBioMed (IAME UMR 1137) and is accessible at http://clermontyping.iame-research.center/.

### Performance assessment

#### On well-characterized strains

To demonstrate the accuracy of ClermonTyping, we gathered a test dataset of 334 well-characterized strains representative of the *E. albertii*, *E. fergusonii*, *Escherichia* clade and *E. coli* phylogroup diversity, encompassing 230 strains from three archetypal collections (ECOR, IAI and NILS) [21–23] as well as 104 archetypal strains [24] from which the complete genomes were available. For all these strains (except SMS-3–5 for which the DNA was not readily available), the species as well as the phylogroup assignment have been determined *in vitro* in our laboratory by the various Clermont PCR assays (Table S2). The sequence type according to the Warwick and Pasteur schemes are indicated when available [9, 10]. Lastly, we undertook a Mash assignment of the strains (Table S2).

**Fig. 4.**
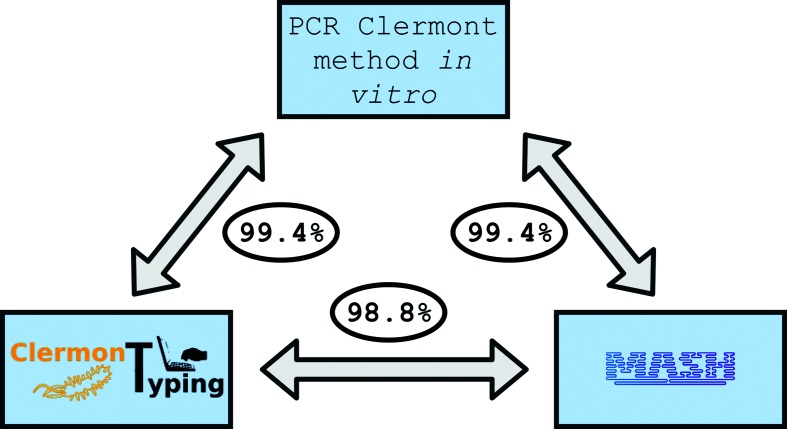
Concordance between the three methods used for *Escherichia* species/phylogroup assignment (PCR Clermont method *in vitro*, ClermonTyper and Mash). The data, presented as percentages, are based on 334 strains representing the *Escherichia* sp./phylogroup diversity (Table S2). The discrepancies between the PCR Clermont method *in vitro* and Mash are due to the limitations of the Clermont method resulting from horizontal gene transfers or SNPs in the primers. The discrepancies between the *in vitro* and *in silico* Clermont methods are due to strain contamination and IS*1* insertion. Finally, concordance between the Mash and *in silico* Clermont methods is subject to the same bias of strain contamination and to the limitations from the original PCR Clermont method cited above.

We then compared the species/phylogroup assignment based on the three methods (*in vitro* PCR, *in silico* PCR and Mash assignment). We observed only two discrepant results between the *in vitro* and *in silico* species/phylogroup typing (strains IAI17 and IAI42), giving an overall concordance above 99 % (Fig. 4). In four cases the Mash phylogroup was not the same as the one given by the *in silico* quadruplex (strains IAI17, IAI24, IAI42 and ECOR44).

We investigated these four cases further and were able to understand the cause of these discrepancies or to provide a probable explanation. Strain IAI17 is determined to be an A phylogroup strain by both *in vitro* PCR (+ − − −) and Mash but appears as a D strain with *in silico* PCR (+ + − −). This is due to the presence of *chuA* in the FASTA file, on a small contig (less than 5 kb) that might be a result of contamination that occurred at some point during the sequencing experiment. This hypothesis was confirmed by re-sequencing of the strain, which provided the expected A phylogroup profile (+ − − −).

Regarding the three other strains, a more biological explanation seems likely. In the case of strain IAI42, the *in silico* method assigned an ‘Unknown’ profile (− − + −) instead of (+ − + −) in the *in vitro* profile (Table S2). In fact, the length of the *arpA* PCR product in the *in vitro* PCR was higher than expected. Classical Sanger sequencing of this PCR product showed the insertion of an IS*1* of 800 bp. The absence of the *arpA* amplicon in the *in silico* method is due to limitation of assembling repeated sequences, making virtual amplification impossible. Re-sequencing of the strain gave the same result. Nevertheless, if the assembly had been possible, the increased length of the virtual amplification of *arpA* would have considered the result as negative. For strains IAI42 and ECOR44, both PCR methods return an E phylogroup because of the *arpA* amplification specific to phylogroup E. If we look at the Mash results, the strains belong to phylogroup D and the same result would be obtained by any method based on whole genome data (Table S2). The origin of this *arpA* allele characteristic of the E phylogroup is probably a horizontal transfer event.

These four complex cases from the whole test dataset show that the method is very robust and gives pointers for the user to determine whether the results should be taken with caution.

#### On other strains found in Enterobase

Of the 334 strains present in Table S2, 311 are *E. coli sensu stricto* and the members of species *E. albertii*, *E. fergusonii* and *E.* clades II–V are under-represented. Because they are far more distant than the *E. coli sensu stricto* strains, they might prove challenging for our method so we gathered a second test dataset consisting only of strains of non-*sensu stricto Escherichia*. These strains were found using Enterobase [25] based on multiple research criteria, resulting in the 180 strains listed in Table S3. For these strains, we achieved 100 % agreement between the *in silico* PCR typing and the Mash typing.

### Updating of the method

The proposed system is based on current knowledge of the *Escherichia* genus phylogeny but can be easily updated. As an example, *E. coli* strains of the ST117 (Warwick nomenclature) lineage appeared as belonging to the F group with both *in vitro* and *in silico* methods. However, these strains are equidistantly located between the B2 and F phylogroups and some authors have recently proposed to create a G phylogroup for this lineage [26]. If this is confirmed using a subsequent panel of strains and a specific gene/SNP is identified, it will be easy to update the ClermonTyper for the assignment of strains to this new phylogroup.

## Conclusion

We have developed ClermonTyping, a user-friendly, open source and freely available method, and its web counterpart, the ClermonTyper, which mimics the *in vitro* PCR assays previously used by the scientific community. This tool allows, with high accuracy, a given strain of the genus *Escherichia* to be assigned to a specific species, and for *E. coli sensu stricto* strains to be assigned to a phylogroup. It has the advantage, in epidemiological studies, to allow comparison with the data obtained using *in vitro* typing assays.

## Supplementary Data

Supplementary File 1

## References

[1] Clermont O, Gordon DM, Brisse S, Walk ST, Denamur E (2011). Characterization of the cryptic *Escherichia* lineages: rapid identification and prevalence. Environ Microbiol.

[2] Walk ST (2015). The "Cryptic" *Escherichia*. EcoSal Plus.

[3] Walk ST, Alm EW, Gordon DM, Ram JL, Toranzos GA (2009). Cryptic lineages of the genus *Escherichia*. Appl Environ Microbiol.

[4] Luo C, Walk ST, Gordon DM, Feldgarden M, Tiedje JM (2011). Genome sequencing of environmental *Escherichia coli* expands understanding of the ecology and speciation of the model bacterial species. Proc Natl Acad Sci USA.

[5] Clermont O, Christenson JK, Denamur E, Gordon DM (2013). The Clermont *Escherichia coli* phylo-typing method revisited: improvement of specificity and detection of new phylo-groups. Environ Microbiol Rep.

[6] Gordon DM, Cowling A (2003). The distribution and genetic structure of *Escherichia coli* in Australian vertebrates: host and geographic effects. Microbiology.

[7] Ingle DJ, Clermont O, Skurnik D, Denamur E, Walk ST (2011). Biofilm formation by and thermal niche and virulence characteristics of *Escherichia* spp. Appl Environ Microbiol.

[8] Tenaillon O, Skurnik D, Picard B, Denamur E (2010). The population genetics of commensal *Escherichia coli*. Nat Rev Microbiol.

[9] Wirth T, Falush D, Lan R, Colles F, Mensa P (2006). Sex and virulence in *Escherichia coli*: an evolutionary perspective. Mol Microbiol.

[10] Jaureguy F, Landraud L, Passet V, Diancourt L, Frapy E (2008). Phylogenetic and genomic diversity of human bacteremic *Escherichia coli* strains. BMC Genomics.

[11] Clermont O, Bonacorsi S, Bingen E (2000). Rapid and simple determination of the *Escherichia coli* phylogenetic group. Appl Environ Microbiol.

[12] Smati M, Clermont O, Bleibtreu A, Fourreau F, David A (2015). Quantitative analysis of commensal *Escherichia coli* populations reveals host-specific enterotypes at the intra-species level. Microbiologyopen.

[13] Lindsey RL, Garcia-Toledo L, Fasulo D, Gladney LM, Strockbine N (2017). Multiplex polymerase chain reaction for identification of *Escherichia coli*, *Escherichia albertii* and *Escherichia fergusonii*. J Microbiol Methods.

[14] Moriel DG, Tan L, Goh KG, Phan MD, Ipe DS (2016). A novel protective vaccine antigen from the core *Escherichia coli* genome. mSphere.

[15] Gangiredla J, Mammel MK, Barnaba TJ, Tartera C, Gebru ST (2017). Species-wide collection of *Escherichia coli* isolates for examination of genomic diversity. Genome Announc.

[16] Kallonen T, Brodrick HJ, Harris SR, Corander J, Brown NM (2017). Systematic longitudinal survey of invasive *Escherichia coli* in England demonstrates a stable population structure only transiently disturbed by the emergence of ST131. Genome Res.

[17] Altschul SF, Gish W, Miller W, Myers EW, Lipman DJ (1990). Basic local alignment search tool. J Mol Biol.

[18] Didelot X, Méric G, Falush D, Darling AE (2012). Impact of homologous and non-homologous recombination in the genomic evolution of *Escherichia coli*. BMC Genomics.

[19] Ondov BD, Treangen TJ, Melsted P, Mallonee AB, Bergman NH (2016). Mash: fast genome and metagenome distance estimation using MinHash. Genome Biol.

[20] Phillippy A, Ondov B (2017). Mash Screen: what’s in my sequencing run?. https://genomeinformatics.github.io/mash-screen/.

[21] Ochman H, Selander RK (1984). Standard reference strains of *Escherichia coli* from natural populations. J Bacteriol.

[22] Picard B, Garcia JS, Gouriou S, Duriez P, Brahimi N (1999). The link between phylogeny and virulence in *Escherichia coli* extraintestinal infection. Infect Immun.

[23] Bleibtreu A, Clermont O, Darlu P, Glodt J, Branger C (2014). The *rpoS* gene is predominantly inactivated during laboratory storage and undergoes source-sink evolution in *Escherichia coli* species. J Bacteriol.

[24] Clermont O, Denamur E, Gordon D (2015). Guide to the various phylogenetic classification schemes for *Escherichia coli* and the correspondence among schemes. Microbiol.

[25] Warwick Medical School (2018). Enterobase. http://enterobase.warwick.ac.uk/.

[26] Lu S, Jin D, Wu S, Yang J, Lan R (2016). Insights into the evolution of pathogenicity of *Escherichia coli* from genomic analysis of intestinal *E. coli* of *Marmota himalayana* in Qinghai-Tibet plateau of China. Emerg Microbes Infect.

